# Reply: Reduced DNA repair in BRCA1 mutation carriers undetectable before onset of breast cancer?

**DOI:** 10.1038/sj.bjc.6603978

**Published:** 2007-09-11

**Authors:** S A Narod

**Affiliations:** 1 Centre for Research in Women's Health, Womens College Hospital, 790 Bay Street, Room 750, Toronto, Ontario, Canada M5G 1N8

**Sir**,

In our study, none of the three tests we used were helpful in distinguishing between BRCA1 mutation carriers and non-carriers. There are three possible explanations: first, this may be because our sample size was small and we had sufficient power to detect only relatively large differences. Second, there may in fact be no phenotype for the BRCA1 heterozygote cell that is discernable in lymphocytes, and cancer predisposition may depend on reduction to homozygosity in a given cell; and third, as Vogel and Surowy point out, our panel of tests may not have been sufficiently sensitive to detect any differences, if they were present. We hope that they are correct and that they, or others, will be successful where we have failed. We remain very keen on using such a test in future intervention trials.

